# Downy Lavender Oil: A Promising Source of Antimicrobial, Antiobesity, and Anti-Alzheimer's Disease Agents

**DOI:** 10.1155/2020/5679408

**Published:** 2020-02-07

**Authors:** Mohammed S. Ali-Shtayeh, Salam Y. Abu-Zaitoun, Nativ Dudai, Rana M. Jamous

**Affiliations:** ^1^Biodiversity and Environmental Research Center (BERC), Til, Nablus, State of Palestine; ^2^Unit of Medicinal and Aromatic Plants, Newe Ya'ar Research Center, Agricultural Research Organization, Ramat Yishay, Israel

## Abstract

*Lavandula pubescens* Decne (LP) is one of the three *Lavandula* species growing wildly in the Dead Sea Valley, Palestine. The products derived from the plant, including the essential oil (EO), have been used in Traditional Arabic Palestinian Herbal Medicine (TAPHM) for centuries as therapeutic agents. The EO is traditionally believed to have sedative, anti-inflammatory, antiseptic, antidepressive, antiamnesia, and antiobesity properties. This study was therefore aimed to assess the in vitro bioactivities associated with the LP EO. The EO was separated by hydrodistillation from the aerial parts of LP plants and analyzed for its antioxidant, antimicrobial, anticholinesterase, and antilipase activities. GC-MS was used for phytochemical analysis. The chemical analysis of the EO composition revealed 25 constituents, of which carvacrol (65.27%) was the most abundant. EO exhibited strong antioxidant (IC_50_ 0.16–0.18 *μ*L/mL), antiacetylcholinesterase (IC_50_ 0.9 *μ*L/mL), antibutyrylcholinesterase (IC_50_ 6.82 *μ*L/mL), and antilipase (IC_50_ 1.08 *μ*L/mL) effects. The EO also demonstrated high antibacterial activity with the highest susceptibility observed for *Staphylococcus aureus* with 95.7% inhibition. The EO was shown to exhibit strong inhibitory activity against *Candida albicans* (MIC 0.47 *μ*L/mL). The EO was also shown to possess strong antidermatophyte activity against *Microsporum canis*, *Trichophyton rubrum*, *Trichophyton mentagrophytes,* and *Epidermophyton floccosum* (EC_50_ 0.05–0.06 *μ*L/mL). The high antioxidant, enzyme inhibitory, and antimicrobial potentials of the EO can, therefore, be correlated with its high content of monoterpenes, especially carvacrol, as shown by its comparable bioactivities indicators results. This study provided new insights into the composition and bioactivities of LP EO. Our finding revealed evidence that LP EO makes a valuable natural source of bioactive molecules showing substantial potential as antioxidant, neuroprotective, antihyperlipidemic, and antimicrobial agents. This study demonstrates, for the first time, that LP EO might be useful for further investigation aiming at integrative CAM and clinical applications in the management of dermatophytosis, Alzheimer's disease, and obesity.

## 1. Introduction

The genus *Lavandula* (Lamiaceae), lavender, is a typical aromatic evergreen understory chamaephyte that comprises about 32 species [1], some of them being utilized in complementary and alternative medicine for a long time, either dried or as essential oils (EOs). Three native *Lavandula* species are growing wild in Palestine (West Bank and Gaza Strip), namely, *L. pubescens* Decne (Downy lavender), *L. stoechas* L. (French lavender), and *L. coronopifolia* Poir. (Staghorn lavender) [2]. *L. pubescens* is common in the Dead Sea Valley, Jerusalem, and Hebron Desert and very rare in the Lower Jordan Valley and *L. coronopifolia* is common only in the Dead Sea Valley and only rare in Jerusalem and Hebron Desert, whereas *L. stoechas* is rare in Gaza Strip. Many pharmacological properties have been reported for lavender EOs, including local anesthetic, sedative, analgesic, anticonvulsant, antispasmodic [3, 4], cholinesterase inhibitory [5], antioxidant [6, 7], antibacterial, and antifungal effects and inhibition of microbial resistance [6, 8], and they are used for the treatment of inflammation and many neurological disturbances [9]. The oil has also been utilized for relieving anxiety and associated sleep disorders [10], depression, and headache [11]. The EO of *Lavandula* species is also used widely in pharmaceutical fragrance, food, and household cleaners [12–14].

The EO of *L. pubescens* has been reported to exhibit a strong wide-ranging *in vitro* antibacterial activity against Gram-positive and Gram-negative bacteria including *Salmonella enterica*, *Staphylococcus aureus*, *Micrococcus luteus*, *Enterococcus faecalis*, and *Escherichia coli* [6, 13, 15] and hepatoprotective [16], cytotoxic, and xanthine-oxidase inhibitory activities [6, 8].

The products derived from the Palestinian Downy lavender (*L. pubescens*) (Arabic, Khuzama), including EO, have been utilized for centuries in Traditional Arabic Palestinian Herbal Medicine (TAPHM) as CAM therapies [17]. The LP EO is traditionally believed to have sedative, anti-inflammatory, antiseptic, antidementia, and antiobesity properties and has therefore been utilized for the management of, but not limited to, indigestion, neurological disorders, dementia, obesity, and microbial skin infections [17].

However, no reports are available on the antidermatophytic, anticholinesterase (i.e., anti-Alzheimer's disease), and antilipase (i.e., antiobesity) effects associated with the EO of *L. pubescens*.

This study was, therefore, aimed at defining the chemical composition of EO attained from above-ground parts of *L. pubescens* plants collected from wild populations in the Dead Sea Valley in Palestine, and assessing its potential in vitro antioxidant, antimicrobial, anticholinesterase, and antilipase effects and thus to verify its use as a complementary medicine for the treatment of AD, obesity, and microbial skin infections.

## 2. Materials and Methods

### 2.1. Plant Material and Essential Oil Extraction

The aerial parts of fully bloomed *Lavandula pubescens* were collected from Palestine (Dead Sea Valley) in May 2017 and used for EO extraction. Plants were authenticated by the first author. The voucher specimen (*Lavandula pubescens* Decne, Voucher No. BERC-BX603) has been deposited at BERC Herbarium, Til, Nablus, Palestine. 250 gm of the fresh above-ground plant parts were subjected to hydrodistillation using a modified Clevenger apparatus until there was no significant increase in the amount of EO collected [18].

### 2.2. GC-MS Analysis of Essential Oil

Gas chromatography-mass spectrometry (GC-MS) was performed to determine the EO composition by using the conditions reported by Ali-Shtayeh et al. [18]. Identification of the compounds was performed by comparing their relative retention indices (RI) with those of authentic compounds (e.g., carvacrol, terpinolene, *ε*-caryophyllene, and *β*-bisabolene) or by comparing their mass spectral fragmentation patterns with Wiley 7 MS library (Wiley, New York, NY, USA) and NIST98 (Gaithersburg, MD, USA) mass spectral database. The identified components along with their RI values and percentage composition are summarized in Table 1.

### 2.3. Antioxidant Activity Evaluation

Antioxidant properties of the EO from *L. pubescens* were evaluated by using the following methods: the 2,2′-azino-bis (3-ethylbenzo thiazoline-6-sulphonic acid) ABTS radical cation decolorization and reductive potential (RP) assays as reported previously [19, 20]. Trolox, ascorbic acid, and BHT were used as standard antioxidants.

### 2.4. Enzymatic Inhibitory Activities

The essential oils of *L. pubescens* and carvacrol were investigated for their enzyme inhibitory properties on acetylcholinesterase (AChE), butyrylcholinesterase (BuChE), and porcine pancreatic lipase (PPL) following previously reported spectrophotometric methods [21, 22]. Neostigmine was used as a reference compound for AChE and BuChE enzymes, and orlistat was used for PPL enzyme.

The effects of different doses of test compounds (LP essential oil, carvacrol and reference compounds) on the AChE, BuChE, and PPL activities were used to calculate the IC_50_ values from dose-effect curves by linear regression.

### 2.5. Microbiological Assays

Microorganisms used in this study are presented in Table 2.

#### 2.5.1. Agar Disc Diffusion Assay

This method was used to evaluate the antimicrobial activities of the EO and carvacrol against *Candida albicans* and bacterial strains as described by the Clinical and Laboratory Standards Institute (CLSI) [23]. The inhibition zone diameter for each sample was measured in mm and used to calculate the antibacterial and anticandidal activity index (AI) and % of inhibition (PI) at a concentration of 1 *μ*L/disc using the following formulas [24]:

(1) $$\begin{array}{c} \text{AI}=\frac{\text{mean zone of inhibition of EO}}{\text{zone of inhibition obtained for standard antibiotic }},\\ \text{PI}=\text{AI}\times 100\%. \end{array}$$

All experiments were done in triplicate. Chloramphenicol and voriconazole were used as positive controls for bacteria and candida, respectively.

#### 2.5.2. Broth Microdilution Assay

The broth microdilution technique with some modifications was used to determine the minimum inhibitory concentration (MIC) values of the EO against bacteria and *C. albicans* strains [25–27]. Chloramphenicol (1 to 64 *μ*g/mL) and voriconazole (0.019 to 1.25 *μ*g/mL) were used as reference antibiotics for bacteria and *Candida*, respectively.

#### 2.5.3. Determination of Antidermatophytic Activity: Poisoned-Food Technique

Essential oils from *L. pubescens* and carvacrol were tested for their antidermatophyte activity against four dermatophytes species: *Microsporum canis*, *Trichophyton mentagrophytes*, *Epidermophyton floccosum,* and *Trichophyton rubrum* (Table 2) using the modified poisoned-food technique [28]. EO and carvacrol were tested at different concentrations (0.5–0.0039 mL/L). Mycelial growth inhibition % (PI) was calculated as follows:

(2) $$\begin{array}{c} \%\text{PI}=\left[\frac{\text{DC}-\text{DT}}{\text{DC}}\right]\times 100, \end{array}$$

where DC is the average diameter of mycelial growth of the control, and DT is the average diameter of mycelial growth of the treatment. Effective concentration fifty (EC_50_) that caused 50% growth inhibition was estimated using Microsoft Excel 2010 under Windows 10.

Minimum inhibitory concentration (MIC) and minimum fungicidal concentration (MFC) were assessed following the previously reported assays [29, 30].

## 3. Results and Discussion

### 3.1. GC-MS Analysis

There are no reports on the EO composition of *L. pubescens* growing wild in Palestine and only a few such reports are available worldwide [6, 8, 13, 31]. Hydrodistillation of the *L. pubescens* leaves yielded 1.9 mL per 250 g fresh plant material.

The GC-MS analysis of the EO led to the identification of 25 components (Table 1). The main identified compounds were carvacrol (65.27%), *β*-bisabolene (7.43%), *ε*-caryophyllene (6.21%), carvacrol methyl ether (5.36%), terpinolene (5.34%), Z-*β*-ocimene (2.63%), myrcene (2.05%), para-menth-1-en-9-ol (1.73%), and caryophyllene oxide (1.11%), representing 97.13% of the total oil. Hence, the EO from the Palestinian *L. pubescens* can be characterized as carvacrol chemotype. The oxygenated monoterpenes were the dominant (73.26%) chemical group within the constituents, followed by sesquiterpene hydrocarbons (13.84%), monoterpene hydrocarbons (11.79%), and oxygenated sesquiterpenes (1.11%). The EO chemical profile in this study is qualitatively comparable to that formerly reported from Yemen where the EO has shown to be carvacrol chemotype (60.9–77.5%) [6, 31].

Carvacrol is a monoterpenic phenol that is biosynthesized from *γ*-terpinene [32] through p-cymene [33]. These two compounds are therefore present in the *L. pubescens* EO. Biosynthetic intermediates such as terpinene-4-ol [34] and p-cymen-8-ol [35] are also present [36].

### 3.2. Antioxidant Potential

The antioxidant activity of EOs is a biological property of great interest because the oils that possess the ability of scavenging free radicals may play an important role in the prevention of some diseases that may result from oxidative stress damages caused by the free radicals, such as brain dysfunction, Alzheimer's disease, obesity, cancer, heart disease, and immune system decline [37–39]. The consumption of naturally occurring antioxidants that can be used to protect human beings from oxidative stress damages has therefore been increased [38]. This work reports the antioxidant activities of *L. pubescens* EO as assessed by ABTS and RP assays (Table 3).

The antioxidant potential of LP EO was generally high with RP_50_ and IC_50_ of 0.16 and 0.18 *μ*L/mL using RP and ABTS assays, respectively. Interestingly, carvacrol has shown comparable antioxidant activity (IC_50_ = 0.03 *μ*L/mL) relative to the potent antioxidant agent BHT using the ABTS assay and high antioxidant capacity (RP_50_ = 0.07 *μ*L/mL) comparable to the tested potent antioxidant agents (Trolox and BHT) (Table 3).

The antioxidant capacities of *L. pubescens* EO may be attributed to the high content of the oil's major phenolic constituents, especially carvacrol, which were confirmed as effective antioxidant compounds with potential health benefits [40]. Our results demonstrate that the EOs of *L*. *pubescens* and carvacrol have a significant strength to provide electrons to reactive oxygen species (ROS), converting them into more stable nonreactive species and ending the free ROS chain reaction.

### 3.3. Antibacterial Activity

**Figure 1:**
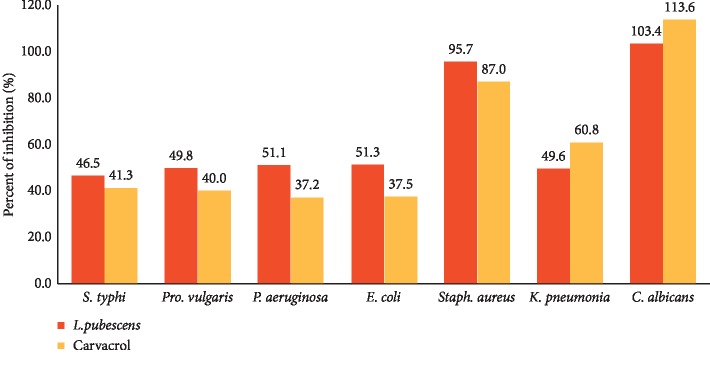
Antimicrobial activity (percent of inhibition) of essential oil and carvacrol on bacteria and *Candida albicans.*

**Figure 2:**
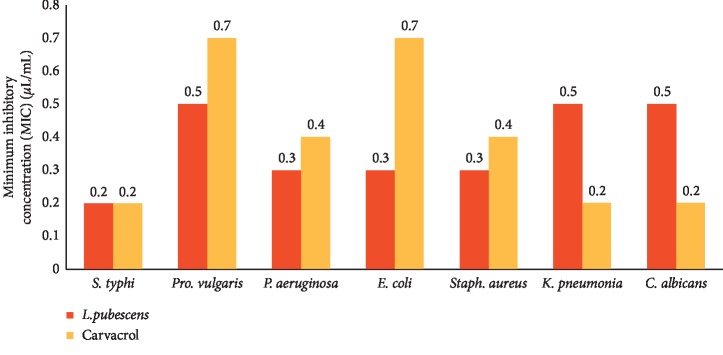
Minimum inhibitory concentration (MIC) values of the essential oil against bacteria strains and *Candida albicans*.

Results for the in vitro antibacterial activity of *L. pubescens* EO and carvacrol are presented in Figures 1 and 2 as PI and MIC. The EO and carvacrol had similar high antibacterial activities against all bacteria tested with a PI range of 37.2–95.7% and MIC range of 0.2–0.7 *μ*L/mL. *Staphylococcus aureus* (Gram-positive) was the most susceptible strain (PI value 95.7% for EO and 87% for carvacrol). Among the tested Gram-negative bacterial strains, the EO has comparable inhibition effect with PI values 46.5, 49.8, 51.1, 51.3, and 49.6% against *Salmonella typhi*, *Proteus vulgaris*, *Pseudomonas aeruginosa*, *E*. *coli*, and *K. pneumonia*, respectively.

The strong antibacterial activity of the EO may be ascribed to the presence of high % of oxygenated monoterpenes (73.26%) such as carvacrol (65.27%), which was found to destroy cell morphology and biofilm viability in typical biofilm construction by increasing the permeability and reducing polarization of the cytoplasmic membrane [41–43]. The antibacterial activity of carvacrol has been mainly attributed to its hydrophobicity and the free hydroxyl group in its structure [44]. With the appropriate hydrophobicity of carvacrol, the compound can be accumulated in the cell membrane, while its hydrogen-bonding and its proton-release abilities may induce conformational modification of the membrane resulting in cell death [45]. Our results can, therefore, explain the association of the use of the LP EO in TAPHM as an antiseptic, due to the antibacterial action of carvacrol which has been previously confirmed [46, 47].

### 3.4. Anticandidal Activity

Candidiasis is a mycotic infection caused by several species of *Candida*, which can endorse superficial and systemic opportunist diseases worldwide. The current treatment against candidiasis is based on synthetic antimycotic drugs. Most presently available anticandidal drugs have limitations that hamper their use, which necessitates the search for safe and effective antimycotic agents.

The results of this study showed that the EO and carvacrol possessed strong inhibitory activity against *C. albicans* (isolated from cutaneous and vulvovaginal infections) with average PI values of 103.4% for EO and 113.6% for carvacrol (Figure 1) and MIC values of 0.47 and 0.24 *μ*L/mL for EO and carvacrol, respectively (Figure 2). The strong anticandidal activity of EO can, therefore, be correlated with its high content of carvacrol owing to the anticandidal activity of carvacrol which has been previously confirmed [48].

### 3.5. Antidermatophytic Activity

**Figure 3:**
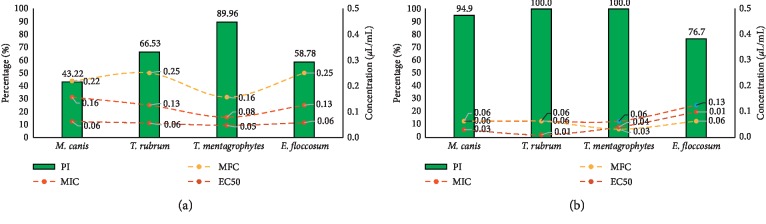
Percentage of mycelial growth inhibition (PI) with MIC, MFC, and EC_50_ values of (a) of *Lavandula pubescens* EO and (b) carvacrol against the tested dermatophytes.

Aromatic plants EOs are known to be mycostatic or fungicidal and represent a potential source of new antimycotics [49]. In view of the increasing resistance to the classical antimycotics, the EOs and their active constituents may be beneficial in the management of mycoses, especially dermatophytosis [50]. In the present study, the *L. pubescens* EO showed strong activity against *M. canis*, *T. rubrum, T. mentagrophytes,* and *E. floccosum* as indicated by their PI, MIC, MFC, and EC_50_ values (Figure 3).

**Figure 4:**
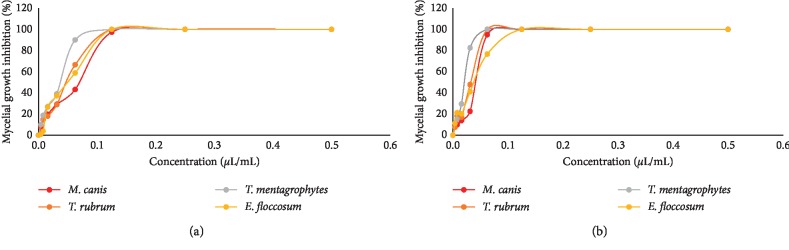
Mycelial growth inhibition activity of (a) *Lavandula pubescens* essential oil and (b) carvacrol against the tested dermatophytes.

The EO of *L. pubescens* and carvacrol showed a dose-dependent activity against the tested dermatophytes (Figure 4). Overall, as the dose of the EO or carvacrol increased, the inhibitory activity against the tested dermatophytes increased indicated by heightened mycelial growth inhibition. The radial mycelial growth of all tested isolates was completely inhibited by the EO and carvacrol at 0.5, 0.25, and 0.125 *μ*L/mL concentration. However, at lower doses (0.004–0.063 *μ*L/mL), the EO was still more active on the mycelial growth of *T. mentagrophytes* than other tested dermatophytes at 0.63 *μ*L/mL, PI = 89.7% (Figure 3).

The MIC and EC_50_ values of the EO of *L. pubescens* on the tested dermatophytes were in the ranges of 0.08–0.16 *μ*L/mL and 0.05–0.06 *μ*L/mL, respectively. However, EO showed a fungicidal effect on the four studied dermatophytes and the MFCs were in the range of 0.16–0.25 *μ*L/mL. *T. mentagrophytes* were more susceptible to *L. pubescens* EO than the other tested fungi with MIC, MFC, and EC_50_ values of 0.05, 0.08, and 0.16 *μ*L/mL, respectively.

The strong antifungal property could be attributed to the major component of the EOs, carvacrol, and the oxygenated monoterpene, which exhibited strong inhibitory activity against the tested dermatophytes (Figure 3) with PI, MIC, EC_50_, and MFC values ranging from 76.7 to 100%, 0.063–0.125 *μ*L/mL, 0.01–0.1 *μ*L/mL, and 0.03–0.63 *μ*L/mL, respectively. The monoterpene alcohols are water soluble and possess functional alcohol groups that explain their strong antidermatophyte activity [49].

In general, EO and carvacrol can exert their antidermatophyte actions due to membrane damage, cytoplasmic content leakage, and ergosterol depletion [49, 51–53].

### 3.6. Enzyme Inhibitory Activities of Essential Oil

#### 3.6.1. Anticholinesterase Activity

Cholinesterase inhibitors (ChEIs) have recently become the most widely used drugs for the management of Alzheimer's disease (AD) [54]. ChEIs play a crucial role in the memory enhancement of AD patients through increasing ACh concentration in neural synaptic clefts and thus improving the brain cholinergic transmission and decreasing *β*-amyloid aggregation and neurotoxic ﬁbrils formation [55–57]. However, synthetic AChEIs including galanthamine and tacrine have restrictions owing to the short half-life and adverse side effects such as digestive disorders, nausea, and dizziness [58, 59]. Hence, it is necessary to explore new safe alternatives with superior characteristics to deal with AD.

Several plants and phytochemical compounds have revealed cholinesterase inhibitory capacity and therefore can be valuable in the management of neurological disturbances [21]. In this study, LP EO was investigated for its in vitro cholinesterases (AChE and BuChE) inhibitory activities. The EO and carvacrol have shown to possess high AChE (IC_50_ = 0.9, and 1.43 *μ*L/mL, respectively) and medium BuChE (IC_50_ 6.82, and 7.75 *μ*L/mL, respectively) inhibitory activities (Table 4).

Thus, the high AChE inhibitory effect of the *L. pubescens* EO in the current study may be mainly associated with its major component, carvacrol, and with its high phenol content. Overall, the tested EO was shown to be more selective inhibitors for acetylcholinesterase than butyrylcholinesterase with a selectivity index (SI) of 7.58.

Our results demonstrate that LP EO could be a valued natural source of AChEIs, e.g., carvacrol, with effective inhibitory activities against the principal enzymes associated with AD and could signify a basis for developing a new treatment strategy for Alzheimer's using plant-derived AChEIs.

#### 3.6.2. Pancreatic Lipase Inhibitory Activity

Pancreatic lipase, the principal enzyme associated with obesity, plays a key role in the efficient digestion of acylglycerols [60]. The hydrolysis of glycerides to glycerol and free fatty acids is performed by lipases. Taking into consideration that 50–70% of the total dietary fat hydrolysis is performed by pancreatic lipase, enzyme inhibition is one of the approaches used to treat obesity [60]. The mechanism involves inhibition of dietary triglyceride absorption, as this is the main source of excess calories [61]. Besides, pancreatic lipase inhibition does not alter any central mechanism, which makes it an ideal approach for obesity treatment [62]. The pancreatic lipase has been widely used for the determination of the potential efficacy of natural products as antiobesity agents [62].

In the present study, *L. pubescens* EO and carvacrol were assessed for their activity against pancreatic lipase. The EO exhibited high inhibitory activity against PPL with IC_50_ of 1.08 *μ*L/mL (Table 5). The high antiobesity activity of *L. pubescens* EO may be mainly ascribed to its high content of carvacrol which has been reported to inhibit visceral adipogenesis and adipocyte differentiation in animal cells and decrease body weight and plasma lipid levels [63, 64]. However, carvacrol on its own cannot explain the high activity of EO, and therefore the totality of constituents of the EO may act synergistically to exert such high antiobesity activity. The higher pancreatic lipase inhibitory effects of *L. pubescens* EO may, therefore, be attributed to its high content of bioactive phenolic acids and flavonoids acting together in a synergistic style [22].

The current study has indicated the ability of the EO to exercise health benefit attributes by inhibiting the pancreatic lipase enzyme (responsible for digestion and absorption of triglycerides) and thus lead to the reduction of fat absorption.

## 4. Conclusions

**Figure 5:**
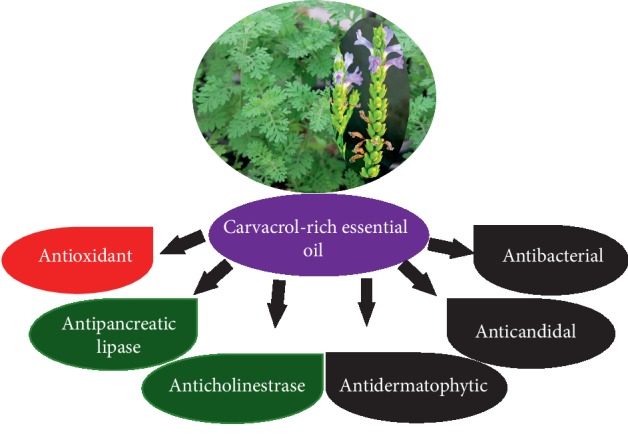
Beneficial health effects of *Lavandula pubecsens* essential oil and its main active constituent, carvacrol.

The main constituent of *L. pubescens* EO was determined as carvacrol in wild plants. The results demonstrate that the plant is a valuable natural source for carvacrol-rich EO with promising potential antimicrobial, antiobesity, and anti-AD health effects (Figure 5). Our results support the use of *L. pubescens* EO as a natural complementary treatment in TAPHM. This is the first report on the antidermatophytic, AChE inhibitory, and antiobesity effects of *L. pubescens* EO. In conclusion, our results might be useful for further investigation aiming at clinical applications of *L. pubescens* EO and carvacrol in the management of AD, obesity, and microbial skin infections including dermatophytosis, candidiasis, and others.

## Figures and Tables

**Table 1:** Chemical composition of the essential oil of *Lavandula pubescens.*

Nu.	Ret time	RI	Compound name	Area %
1	6.93	988	Myrcene	2.05
2	7.383	1002	*α*-Phellandrene	0.14
3	7.456	1008	3-*δ*-Carene	0.20
4	7.681	1014	*α*-Terpinene	0.15
5	7.89	1022	p-Cymene	0.20
6	8.03	1029	Limonene	0.12
7	8.104	1026	1,8-Cineole	0.05
8	8.225	1032	Ζ-*β*-Ocimene	2.63
9	8.519	1044	Ε-*β*-Ocimene	0.20
10	9.667	1086	Terpinolene	5.34
11	9.781	1089	p-Cymenene	0.10
12	10.068	1054	*α*-Terpinolene	0.04
13	10.439	1108	1,3,8-p-Menthatriene	0.03
14	12.631	1179	p-Cymen-8-ol	0.53
15	12.874	1186	4-Terpineol	0.21
16	13.029	1201	4,5-Epoxy-1-isopropyl-4-methyl-1-cyclohexene	0.36
17	13.308	1215	2,6-Dimethyl-3,5,7-octatriene-2-ol	0.08
18	14.158	1241	Carvacrol methyl ether	5.36
19	15.695	1286	Thymol	0.26
20	16.071	1298	Carvacrol	65.27
21	16.082	1294	Para-menth-1-en-9-ol	1.73
22	19.241	1417	*ε*-Caryophyllene	6.21
23	20.172	1452	*α*-Humulene	0.20
24	21.544	1505	Β-Bisabolene	7.43
25	23.387	1582	Caryophyllene oxide	1.11

**Table 2:** Test microorganisms.

Microorganisms	Species name	Source	Notes
Bacteria	*Staphylococcus aureus*	ATCC 25923	Gram positive
	*Proteus vulgaris*	ATCC 13315	Gram negative
	*Pseudomonas aeruginosa*	ATCC 27853	
	*Salmonella typhi*	ATCC 14028	
	*Escherichia coli*	ATCC 25922	
	*Klebsiella pneumonia*	ATCC 13883	

*Candida*	*Candida albicans*	CBS6589	
		CBS9120	
		BERC M77	Clinical isolates (vulvovaginal and cutaneous candidiasis patients)
		BERC N17	
		BERC N40	

Dermatophytes	*Microsporum canis*	CBS 132.88	
		BERC MC03	Clinical isolates (dermatophytosis patients)
		BERC MC39	
		BERC MC13	
	*Trichophyton rubrum*	BERC CBS 392.58	
		BERC TR64	Clinical isolates (dermatophytosis patients)
		BERC TR67	
		BERC TR69	
	*Trichophyton mentagrophytes*	CBS 106.67	
		BERC TM1	Clinical isolates (dermatophytosis patients)
		BERC TM2	
		BERC TM78	
	*Epidermophyton floccosum*	CBS 358.93	

**Table 3:** Antioxidant activities of essential oil from aerial parts of Lavandula pubescens.

	ABTS	Reductive potential
	*IC*_*50*_ *(μL/mL)*	
Oil	0.18 ± 0.05	0.16 ± 0.0
Carvacrol	0.03 ± 0.0	0.07 ± 0.0

	*Standard antioxidants IC*_*50*_ *(mg/ml)*	
Trolox	0.05 ± 0.0	0.08 ± 0.0
Ascorbic acid	0.05 ± 0.0	0.04 ± 0.0
BHT	0.03 ± 0.0	0.07 ± 0.01

**Table 4:** Cholinesterase inhibitory activity (ChEIA) of *L. pubescens* essential oil.

	IC_50_ (*μ*L/mL)		Selectivity index (SI)^*∗*^
	Acetylcholinestrase	Buterylcholinestrase	
Oil	0.9 ± 0.14	6.82 ± 0.35	7.58 ± 0.13
Carvacrol	1.43 ± 0.56	7.75 ± 0.25	5.42 ± 0. 01
Neostagmin (*μ*g/mL)	1.54 ± 0.00	174.41 ± 0.00	113.18 ± 0.00

^*∗*^SI = IC_50_ BuChE/IC_50_ AChE.

**Table 5:** Antiobesity activities of *Lavandula pubescens* essential oil.

	IC_50_ (*μ*L/mL)
Oil	1.08 ± 0.35
Carvacrol	6.63 ± 1.03
Orlistat (*μ*g/ml)	0. 12 ± 0.03

## Data Availability

The data used to support the findings of this study are available from the corresponding author upon request.

## Abbreviations

ABTS: 2,2′-Azino-bis (3-ethylbenzo thiazoline-6-sulphonic acid)
AChE: Acetylcholinesterase
AD: Alzheimer's disease
AI: Activity index
BERC: Biodiversity and environmental research center
BuChE: Butyrylcholinesterase
CLSI: Clinical and Laboratory Standards Institute
EC_50_: Effective concentration fifty
EO: Essential oil
GC-MS: Gas chromatography-mass spectrometry
IC_50_: Inhibitory concentration fifty
MFC: Minimum fungicidal concentration
MIC: Minimum inhibitory concentration
PI: Percent of inhibition
PPL: Porcine pancreatic lipase
ROS: Reactive oxygen species
RP: Reductive potential
TAPHM: Traditional Arabic Palestinian herbal medicine.
